# Environment and host species shape the skin microbiome of captive neotropical bats

**DOI:** 10.7717/peerj.2430

**Published:** 2016-09-20

**Authors:** Virginie Lemieux-Labonté, Nicolas Tromas, B. Jesse Shapiro, François-Joseph Lapointe

**Affiliations:** Département de Sciences Biologiques, Université de Montréal, Montréal, Canada

**Keywords:** Bat skin microbiome, Habitat–microbiome interaction, Host–microbiome interaction

## Abstract

**Background:**

A wide range of microorganisms inhabit animal skin. This microbial community (microbiome) plays an important role in host defense against pathogens and disease. Bats (Chiroptera: Mammalia) are an ecologically and evolutionarily diversified group with a relatively unexplored skin microbiome. The bat skin microbiome could play a role in disease resistance, for example, to white nose syndrome (WNS), an infection which has been devastating North American bat populations. However, fundamental knowledge of the bat skin microbiome is needed before understanding its role in health and disease resistance. Captive neotropical frugivorous bats *Artibeus jamaicensis* and *Carollia perspicillata*provide a simple controlled system in which to characterize the factors shaping the bat microbiome. Here, we aimed to determine the relative importance of habitat and host species on the bat skin microbiome.

**Methods:**

We performed high-throughput 16S rRNA gene sequencing of the skin microbiome of two different bat species living in captivity in two different habitats. In the first habitat, *A. jamaicensis* and *C. perspicillata* lived together, while the second habitat contained only *A. jamaicensis.*

**Results:**

We found that both habitat and host species shape the composition and diversity of the skin microbiome, with habitat having the strongest influence. Cohabitating *A. jamaicensis* and *C. perspicillata* shared more similar skin microbiomes than members of the same species (*A. jamaicensis*) across two habitats.

**Discussion:**

These results suggest that in captivity, the skin microbial community is homogenised by the shared environments and individual proximities of bats living together in the same habitat, at the expense of the innate host species factors. The predominant influence of habitat suggests that environmental microorganisms or pathogens might colonize bat skin. We also propose that bat populations could differ in pathogen susceptibility depending on their immediate environment and habitat.

## Introduction

Animal skin is an ecosystem inhabited by highly variable and complex communities of microorganisms (Grice & Segre, 2011), which can be divided into resident and transient flora acquired from the environment (Roth & James, 1988). A healthy skin microbiome contributes to host fitness by occupying pathogen adhesion sites and producing pathogen inhibitors (Roth & James, 1988; Grice & Segre, 2011). For example, the salamander skin associated bacteria *Janthinobacterium lividum* confers resistance to the devastating fungal pathogen *Batrachochytrium dendrobatidis* (Brucker et al., 2008), possibly explaining why some salamander populations decline while others do not. Competitive interactions between beneficial and pathogenic skin microbes may therefore play a role in preventing disease in wild animals (Belden & Harris, 2007). Despite its importance, little is know about the factors shaping the skin microbiome of wild animal species.

Exogenous (environmental) and endogenous (host-specific) factors such pH, sebum production, temperature and moisture (Grice & Segre, 2011) are known to shape the skin microbiome, but the relative influence of these parameters differ between studies. In the gut microbiome, host taxa and phylogeny appears to have a greater effect than the environment on the assemblage of bacterial communities (Ochman et al., 2010; Roeselers et al., 2011; Tzeng et al., 2015). In primates, Moeller et al. (2013) concluded that sympatric members of different species (i.e., gorillas and chimpanzees sharing the same habitat) harbor a more similar gut microbiome than allopatric members of the same species. In neotropical bats, gut microbiomes have been proposed to be influenced by a complex interaction between exogenous and endogenous factors (Phillips et al., 2012), with an important role for host taxa and phylogeny (Phillips et al., 2012; Carrillo-Araujo et al., 2015).

Due to its direct exposure to the environment, the skin microbiome is suspected to be much more dynamic than the gut microbiome (Romano-Bertrand et al., 2015). Hence, the role of the environment is expected to be strong in shaping the skin microbiome. Studies of the skin microbiome of wild populations of amphibians suggest that host species does play a major role because the skin microbiomes of cohabitating species were found to be significantly different (McKenzie et al., 2012; Kueneman et al., 2014; Walke et al., 2014). However, Kueneman et al. (2014) identified the habitat as the second most important parameter on the skin microbiome of amphibian species. Indeed, the environment should act as a bacterial reservoir for the skin microbiome and host species may be able to select particular taxa (Loudon et al., 2014; Walke et al., 2014). Consequently, it is thought that host and environmental factors interact closely to shape the amphibian skin microbiome.

In spite of recent investigations on the skin microbiome of various animal species, few studies have analyzed the relative influence of endogenous (host) and exogenous (environmental) factors on the skin microbiome in non-human mammals. In humans, different variables are expected to shape the skin microbiome, such as body site, age, gender and habitat (Fierer et al., 2008; Giacomoni, Mammone & Teri, 2009; Ying et al., 2015). Humans were found to share more similar microbiomes with their dogs than with dogs from different households (Song et al., 2013). Therefore, a shared habitat might homogenize skin microbiomes across individuals and even across species.

In contrast to the gut microbiome, the skin microbial community appears to be much more influenced by exposure to the environment, including environmental microbes and abiotic factors (Cheng et al., 2015). Yet, understanding the complexity of the skin microbiome clearly suffers from a lack of studies across different mammals. Bats (Chiroptera: Mammalia) are part of one of the most ecologically and evolutionarily diversified mammalian orders (Kunz & Fenton, 2003; Voigt & Kingston, 2016), providing an excellent model to study how microbiomes vary across related host species. As the only flying mammals, with a cave-dwelling lifestyle, the skin microbiome of bats is probably unique among all vertebrates. Additionally, the skin microbiome provides a possible defense against white nose syndrome (WNS), a skin disease caused by the fungus *Pseudogymnoascus destructans* (*Pd*) (Gargas et al., 2009; Lorch et al., 2011), formerly known as *Geomyces destructans*; (Minnis & Lindner, 2013) and responsible for the death of over 6 million North American bats (US Fish & Wildlife Service, 2012). Not all bat species are equally affected by the disease (Turner, Reeder & Coleman, 2011), suggesting that both host genetics, ecology, and microbiomes might play a role in *Pd* resistance. In the light of this knowledge, basic information about fundamental sources of variations in the bat skin microbiome is badly needed to harness the possible implications of this microbial community in a disease context.

Jamaican fruit bats (*Artibeus jamaicensis*) and Seba’s short-tailled bats (*Carollia perspicillata*) provide convenient animal models to study the skin microbiome of chiropterans. These species of neotropical bats (family Phyllostomidae) are widely distributed in Central and South America. They share a gregarious lifestyle, with a polygynous (harem) social organisation based on male defense of the roosting sites where females aggregate (Porter, 1978; Williams, 1986; Ortega & Arita, 1999). In the wild, both species roost in hollow trees and caves (Morrison, 1979; Williams, 1986; Cloutier & Thomas, 1992; Ortega & Arita, 1999), where *A. jamaicensis* normally aggregate in small groups (<12 individuals) or very large colonies (>500 bats) (Arita & Vargas, 1995), and *C. perspicillata* aggregate in groups of 10 to more than 100 individuals (Cloutier & Thomas, 1992). These species are generalist frugivores (Cloutier & Thomas, 1992; Ortega & Castro-Arellano, 2001). They are easily maintained in captivity, where they can even breed. The gut microbiome of both species have recently been characterized (Carrillo-Araujo et al., 2015), while the skin microbial community still remains unknown at this date.

Here, we studied how habitat factors (including colony parameters and diet) and host species contributed to the structure of *A. jamaicensis* and *C. perspicillata* skin microbiome under stable environmental conditions (i.e., in captivity). Although such tropical species are not affected or endangered by the white nose syndrome, they provide a useful model to study the factors that shape the skin microbiome and might provide resistance to pathogens. The skin microbiomes of wild and captive organisms are certainly different (Becker et al., 2014; Loudon et al., 2014; Cheng et al., 2015), but studying them in captivity is practical, allowing us to limit environmental fluctuations that might obscure the effects of host species in natural setting. We used high-throughput 16S amplicon sequencing to assess the taxonomic composition and diversity of the skin microbiome of these two species of bats sampled in two different zoos (habitats). This design allowed us to compared bat groups living in shared vs. separate environments. The objectives were to quantify the contributions of habitat and host species in shaping the bat skin microbiome. Our results show a significant effect of both habitat and host species on the skin microbiome, with habitat playing a dominant role. This study thus provides an initial view of what factors shape the skin microbiome of neotropical bats. These findings provide basic knowledge of the skin microbiome, which can ultimately be applied to the management and conservation of threatened bat populations.

## Materials and Methods

### Sampling

In this study, 42 adult specimens from two different frugivorous bat species were sampled in two different zoos. Namely, 10 *A. jamaicensis* and 12 *C. perspicillata* individuals (all males) were sampled in December 2014 from the Montréal Biodôme (Canada), where they live together in an artificial cave maintained at a temperature of 22 °C during winter, and 26 °C in summer. In addition, 20 *A. jamaicensis* individuals (6 males and 14 females) were also sampled in March 2015 from the Granby Zoo (Canada). These bats also live in an artificial cave, where the temperature is maintained at 26 °C all year long. Both colonies of bats were established in 1992, with actual population sizes of 95 individuals at the Montréal Biodôme (45 *A. jamaicensis* and 50 *C. perspicillata*), and 247 individuals at the Granby Zoo.

Skin microbiome samples were obtained from each specimen by swabbing the back and one wing for 30 s with a sterile Whatman Omniswab (Fisher Scientific) soaked in NaCl 0.15 M. Swabs tips were ejected into Mobio Powersoil DNA isolation Kit tubes (MoBio Laboratories), which were then stored at −20 °C until DNA extraction. As a negative control, a humidified sterile swab was also collected at each sampling site. Handling of animals at the Granby Zoo (as well as the Montréal Biodôme) was approved by the local ethics committees (Comité Opérations en Conservation et Recherche, and Biodôme’s Welfare Animal and Ethics committee).

### DNA extraction, amplification and sequencing

Bacterial genomic DNA was extracted from each swab using the MoBio Powersoil DNA isolation Kit according to the manufacturer’s protocols. Amplification and sequencing were then performed as previously described (Preheim et al., 2013a). Libraries were prepared using a two-step PCR. The first PCR amplifies the hypervariable region V4 of the 16S small subunit ribosomal gene with forward primer U515_f: ACACGACGCTCTTCCGAT CTYRYRGTGCCA GCMGCCGCGGTAA and reverse primer E786_R: CGGCATTCCTG CTGAACCGCTCTTCC GATCTGGACTACHVGGGTWTCTAAT (Caporaso et al., 2011). 2 µl of extracted DNA (equivalent DNA amount by sample) was added to the PCR reaction containing 14.25 µl of sterile water, 5 µl HF buffer, 0.5 µl DNTPs, 0.25 µl Phusion High-Fidelity DNA Polymerase (New England Biolabs inc.), and 1.5 µl of forward and reverse primers. Amplifications were performed with a Mastercycer nexus GSX1 (Eppendorf) under the following conditions: initial denaturation at 98 °C for 30 s; 30 cycles alternating 98 °C for 25 s, 40 s at 54 °C, 35 s at 72 °C, and final elongation step for one minute at 72 °C. Negative controls were included in the amplification step to account for possible contamination. Each sample was amplified in quadruplicate and pooled to limit possible PCR artefacts. All PCR products were then purified with ZYMO DNA Clean & Concentrator™-5 (ZYMO RESEARCH) following the manufacturer’s protocol. The second PCR step consisted of adding primers containing a barcode (index) and Illumina adapter sequences to each DNA amplicon. To do so, 4 µl of the first step amplification product was added to a PCR reaction containing 10.25 µl of sterile water, 5 µl HF buffer, 0.5 µl DNTPs, 0.25 µl Phusion High-Fidelity DNA Polymerase and 2.5 µl of forward primer PE-III-PCR-F: AATGATACGGCGACCACCGAGATCTACACTCTTTCCCTAC ACGACGCTCTTCCGATCT and reverse primer PE-III-PCR-001-096: CAAGCAGA AGACGGCATACGAGATNNNNNNNNNCGGTCTCGGCATTCCTGCTGAACCGCT CTTCCGATCT (N indicating the unique barcode) (Preheim et al., 2013b). Indexing was performed under the following thermal conditions: initial denaturation at 98 °C for 30 s, 7 cycles alternating 98 °C for 30 s, 30 s at 83 °C, and finally 30 s at 72 °C. This second amplification was performed in triplicate. Samples were pooled and purified with the PCR purification Agencourt AMPure XP (Beckman Coulter). Qubit 2.0 Fluorometer (Invitrogen) was used to measure DNA concentration in each sample. Indexed samples were then pooled to obtain a final concentration range between 10 and 20 ng/µl. DNA was next diluted and denatured according to the manufacturer’s protocol for paired-end sequencing using MiSeq Reagent Kit v2 (500 cycles) 2 × 250 bp on MiSeq (Illumina).

### Data analysis

2,105,588 sequences were amplified from 41 of the 42 skin samples. One specimen of *A. jamaicensis* from Granby Zoo was removed from the data set due to failure of sequencing. A mean of 51,356 sequences was obtained per sample, with a minimum of 2,247 and a maximum of 243,588 sequences. Raw sequence data and metadata are available on Figshare at DOI: 10.6084/m9.figshare.3206668 and DOI: 10.6084/m9.figshare.3428159.

Preclustering, quality filtering, primer removal, merging of raw sequences, and postclustering dereplicating were performed with the SmileTrain scripts (https://github.com/almlab/SmileTrain/wiki/) for 16S data processing using USEARCH v. 7.0.1090 (http://www.drive5.com/usearch/) (Edgar, 2010). Distribution-based clustering (Preheim et al., 2013b) using the dbOTUcaller algorithm (https://github.com/spacocha/dbOTUcaller) was performed to cluster sequences into Operational Taxonomic Units (OTUs) by considering the distribution of DNA sequences across samples and sequence distances. The corresponding OTU table providing relative abundances of bacterial taxa in the different samples was assigned with QIIME version 1.8. (http://qiime.org/) (Caporaso et al., 2010) using GreenGenes database release 13_5 (http://greengenes.lbl.gov) (DeSantis et al., 2006) (see Table S1). For compositional analysis, the genus *Halomonas*, *Shewanella* and *Lactobacillus* were identified as contamination because of their high proportion in negative controls. These taxa were consequently filtered out from all samples prior to further analysis.

The Linear Discriminant Analysis (LDA) size Effect (LEfSe) algorithm (https://huttenhower.sph.harvard.edu/galaxy/) (Segata et al., 2011) was used to identify taxa and OTUs contributing the most to differences between habitats and host species. LEfSe detects significant differences in taxa and OTU abundance with the non-parametric factorial Kruskal-Wallis sum rank test (Kruskal & Wallis, 1952). Then, a canonical method is applied to estimate linear combinations of OTUs that provide the best discrimination among bat species or habitats.

To investigate the diversity of the skin microbial community (alpha diversity), Shannon (Hill, 1973) and Balanced Weighted Phylogenetic Diversity (BWPD) (Barker, 2002; Vellend et al., 2011; McCoy & Matsen IV, 2013) indices were computed from multiple rarefied data sets. Multiple rarefaction consists of a repeated subsampling of the OTU table. This procedure is generally used to ensure a more consistent comparison between samples in which the number of sequences differs. Fifty iterations of the deepest sequencing depth for each sample were used in alpha diversity calculations. The Shannon index, which includes both OTU richness and evenness, was computed due to its reduced sensitivity to sample depth differences (Haegeman et al., 2013; Preheim et al., 2013a). BWPD is a diversity measure that uses phylogenetic information to evaluate diversity of microbial community where species delimitation is difficult. Contrary to the Phylogenetic Diversity (PD) measure, BWPD accounts for abundance and is robust to sampling depth differences between samples (McCoy & Matsen IV, 2013). R version 3.1.3 (http://www.r-project.org/) (R Developement Core Team, 2015) was used for all statistical analyses. Alpha diversity results were compared between habitats and species using non-parametric Wilcoxon Signed-Rank test (Wilcoxon, 1945). The *p*-value for all tests was adjusted with Holm’s sequential Bonferroni (Holm, 1979). Phylogenetic diversity indices were calculated using a phylogenetic tree constructed with FastTree 2.1.8 (http://meta.microbesonline.org/fasttree/) (Price, Dehal & Arkin, 2010).

Beta diversity was calculated between bat microbiomes grouped according to habitat and host species. Phylogeny-based weighted UniFrac distances (Lozupone & Knight, 2005; Lozupone et al., 2007) and the square root of Jensen–Shannon divergence (JSD^1/2^) (Fuglede & Topsøe, 2004) were calculated on unrarefied data as previously suggested (McMurdie & Holmes, 2014) with the phyloseq package (https://joey711.github.io/phyloseq/) (McMurdie & Holmes, 2013). Weighted UniFrac, which accounts for differences in abundance, is widely used to compare distances between microbial communities, although it is sensitive to differences in sequencing depth between samples (Lozupone et al., 2011). To address this problem, we used relative OTU abundances to calculate weighted UniFrac distances (McMurdie & Holmes, 2014). JSD was also selected because it is a robust measure of divergence based on the distribution of relative abundances between microbial communities. Taking the square root of JSD transforms this measure into an interpretable metric (Preheim et al., 2013a). All beta diversity results were visualized with non-metric multidimensional scaling (NMDS) (Kruskal, 1964) using the phyloseq *ordinate()* function.

To test for significant differences among groups of bats, we used the permutational multivariate analysis of variance (PERMANOVA), an analog of MANOVA for partitioning distance matrices among various sources of variation (Anderson, 2001). The null hypothesis of this test is that the metric centroid does not differ between groups (in our case, host species and habitat) (Anderson & Walsh, 2013). PERMANOVA was calculated with the *adonis*() function in the vegan package (http://cran.r-project.org/package=vegan) (Oksanen et al., 2015). Since this test is sensitive to data dispersion and may therefore confuse within-group variation with among-group variation (Anderson, 2001), we performed an analysis of multivariate homogeneity (PERMDISP) (Anderson, 2006) with the *betadisper()* function to test if groups differed in their dispersion. The null hypothesis of this test is that the average within-group dispersion is the same in all groups (Anderson & Walsh, 2013). In each of these two tests, the number of permutations was set to 9999. For all analyses, a *p*-value threshold of 0.05 was considered significant.

## Results

### Habitat and host species both shape the composition of bat skin microbiomes

**Figure 1:**
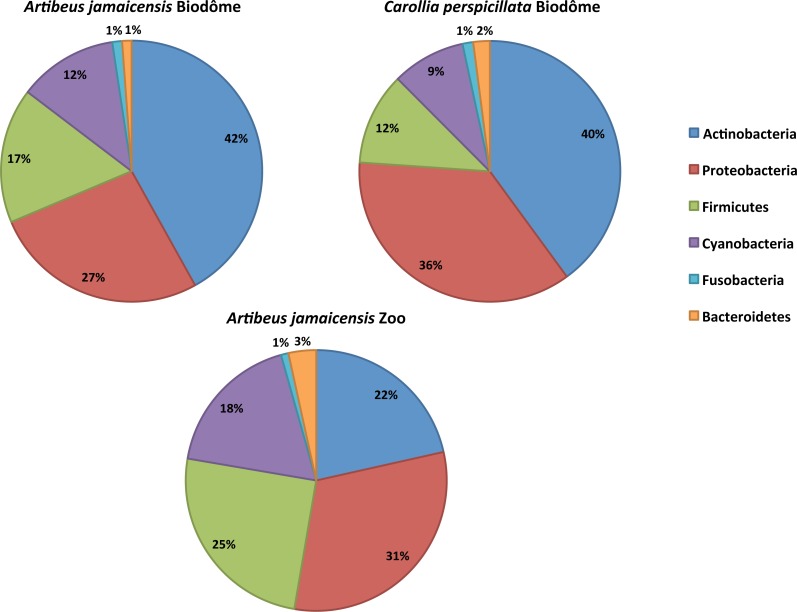
Relative abundances of the six dominant bacterial phyla in the skin microbiome of captive neotropical bats. The complete list of taxa is provided in Table S2.

**Figure 2:**
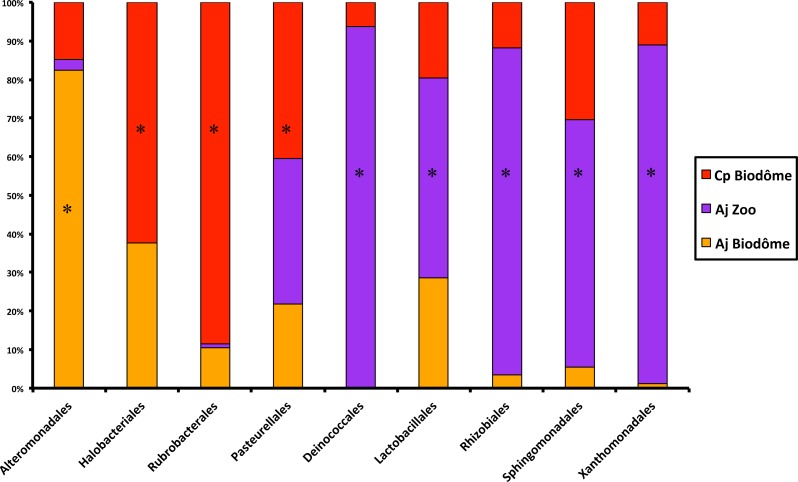
Results of LEfSe analysis showing the main differences among bacterial orders in the skin microbiome of captive neotropical bats. Significant results are identified with a star (*). See ‘Methods’ for more details. Cp Biodôme, Biodôme *C. perspicillata*; Aj Zoo, Granby Zoo *A. jamaicensis*; Aj Biodôme, Biodôme *A. jamaicensis*.

We first characterized the taxonomic composition of the bat skin microbiome by sequencing skin swabs from 41 captive bats. We identified five dominant shared phyla in the skin microbiome of captive bats (Fig. 1): Actinobacteria (22%–42%), Proteobacteria (27%–36%), Firmicutes (12%–25%), Cyanobacteria (9%–17%), Bacteroidetes (1%–3%) and Fusobacteria (∼1%). At the order level, LEfSe analysis nine taxa that differed significantly by either host species or habitat (LDA score ≥ 3.4, *p* < 0.05). Namely, five taxa were representative of *A. jamaicensis* from the Granby Zoo, whereas one and three taxa were respectively representative of *A. jamaicensis* and *C. perspicillata* from the Biodôme (Fig. 2). According to these results, *A. jamaicensis* sampled from the Granby Zoo appears to be the most different group in terms of differentially abundant taxa.

**Figure 3:**
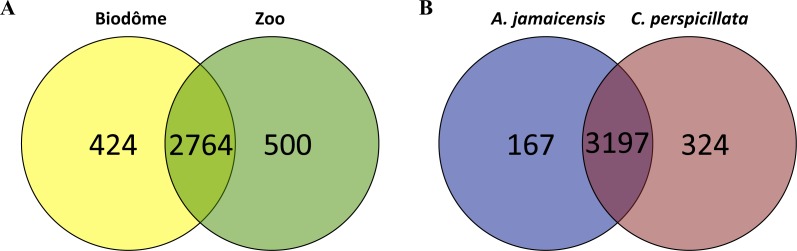
Number of OTUs (operational taxonomic units) enriched in different habitats or host species. (A) Representative OTUs according to habitat (Biodôme or Granby Zoo). (B) Representative OTUs according to bat species (*A. jamaicencis* or *C. perspicillata*). The intersection indicates the numbers of OTUs that did not differ significantly between groups by LEfSe analysis (Methods).

At finer taxonomic resolution, a LEfSe analysis at the OTU level also revealed the importance of habitat in shaping the skin microbiome composition. We identified 924 OTUs significantly enriched in a particular habitat (Fig. 3A)—almost twice the number of OTUs enriched in a particular host species (Fig. 3B). These results suggest that habitat plays a stronger role than host species in shaping the skin microbiome.

### Habitat is a major determinant of alpha diversity

**Figure 4:**
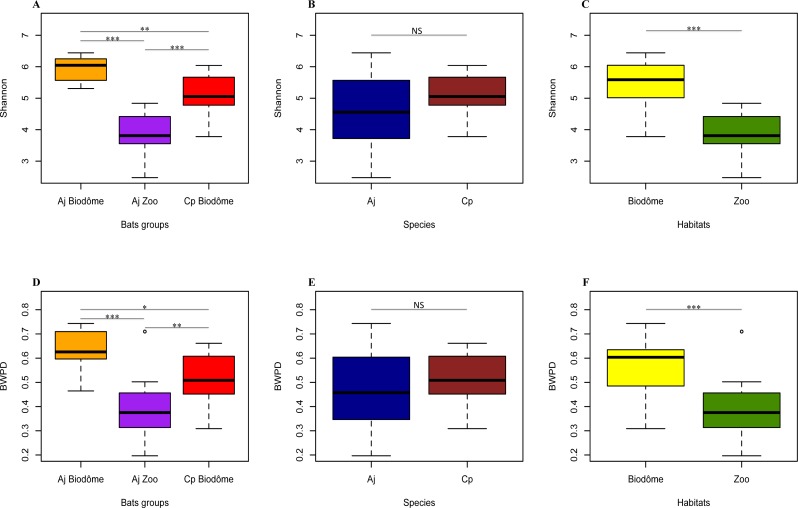
Alpha diversity differs significantly by habitat. (A) Shannon index compared across bat groups. (B) Shannon index compared across bat species. (C) Shannon index compared across habitats. (D) BWPD index (phylogenetic measure) across bat groups. (E) BWPD index across bat species. (F) BWPD index across habitats. Error bars represent standard deviations. Aj Biodôme, Biodôme * A. jamaicensis*; Aj Zoo, Granby Zoo *A. jamaicensis*; Cp Biodôme, Biodôme *C. perspicillata*. Non-parametric Wilcoxon Signed-Rank test ^∗^*p* ≤ 0.05, ^∗∗^*p* ≤ 0.01, ^∗∗∗^*p* ≤ 0.001.

We next asked whether the total amount of diversity (alpha diversity) in the bat skin microbiome differed according to host species or habitat (Fig. 4). Based on the Shannon index of alpha diversity, we found that *A. jamaicensis* from Biodôme is most diverse, and *A. jamaicensis* from Granby Zoo is least diverse (Fig. 4A). Thus, the two Biodôme species seems to harbor a more rich and even skin microbiome community. Shannon diversity between species *A. jamaicensis* and *C. perspicillata* is not significantly different (Fig. 4B, Wilcoxon Signed-Rank test, *V* = 227, *p* = 0.134), while the Biodôme bats (of both species) have significantly higher diversity than Granby Zoo bats (Fig. 4C, Wilcoxon Signed-Rank test, *V* = 400, *p* < 0.001).

The results based on Shannon diversity were confirmed by BWPD, another measure of alpha diversity, which accounts for the phylogenetic relatedness and relative abundance of microbial taxa (Fig. 4D). As observed with Shannon diversity, *A. jamaicensis* from Biodôme has the highest alpha diversity, followed by *C. perspicillata* from Biodôme and *A. jamaicensis* from Granby Zoo (Fig. 4D). The BWPD index is not significantly different between bats species (Fig. 4E, Wilcoxon Signed-Rank test, *V* = 211, *p* = 0.300), whereas significant differences exist between bats sampled from the Biodôme and Granby Zoo habitats (Fig. 4F, Wilcoxon Signed-Rank test, *V* = 363, *p* < 0.001). Results of the BWPD and Shannon index are thus consistent and suggest habitat (and possibly a habitat-species interaction) as the principal forces shaping alpha diversity in the skin microbiome community.

### Habitat and host species shape microbial community beta diversity

**Figure 5:**
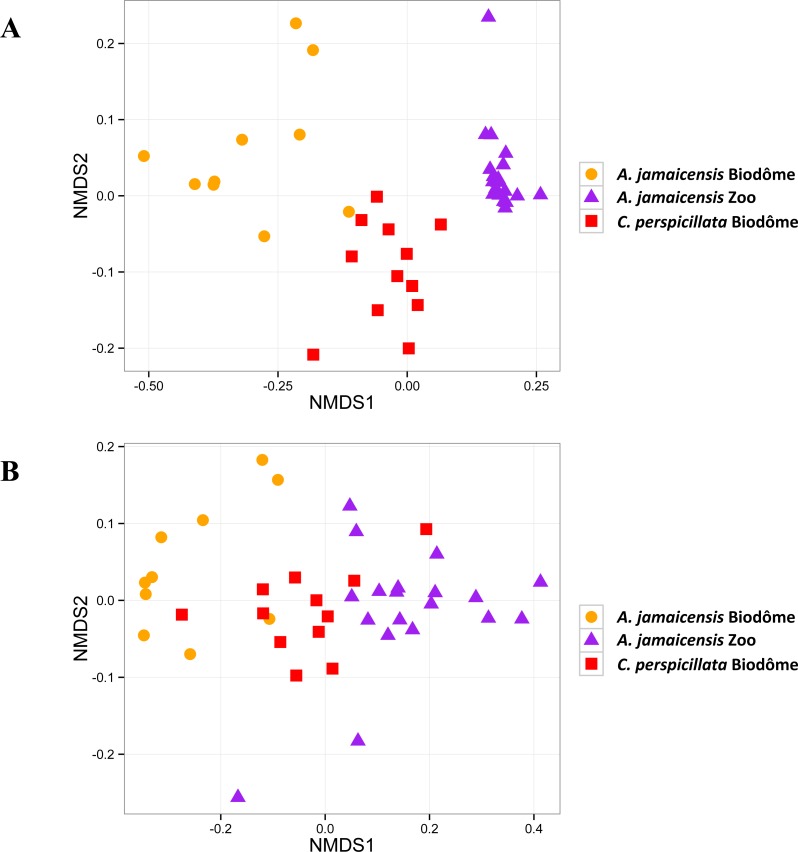
Microbiomes cluster mainly by habitat, but also by host species. (A) Non-metric multidimentional scaling of JSD^1/2^ of bat skin microbiome composition. Each point represents a single microbiome sample. 2D stress = 0.09. (B) Non-metric multidimentional scaling of weighted UniFrac distances among bat skin microbiomes. 2D stress = 0.09.

We next used beta diversity analysis to estimate the effects of the habitat and host species in shaping the composition of the skin microbiome. Our results show that samples are clustered by both habitat and host species, based on two different metrics of beta diversity (Fig. 5), and that all samples are clearly distinct from negative controls (Fig. S1). Using the JSD^1/2^ beta diversity index, individuals of different species from the same habitat (Biodôme) appear to be more closely clustered than individuals of the same species (*A. jamaicensis*) from distinct habitats (Fig. 5A). The weighted UniFrac analysis still discriminated the samples by habitat at the expense of host species (Fig. 5B). Ordination of only Granby Zoo, which included both male and female * A. jamaicensis* bats, does not show any clustering according to sex (data not shown). However our limited sample size (only 6 males) prevents us from drawing firm conclusion on the influence of sex on the bat skin microbiome. Sex therefore remains a possible confounding factor of habitat, because the Biodôme contains all male bats, where the Granby zoo was predominantly female. Globally, beta diversity ordinations thus suggest a predominant influence of habitat on skin microbiome beta diversity.

PERMANOVA analyses also strongly supported the ordination results. Habitat and host species together explained the most variation in JSD^1/2^ (46.04%, *adonis*, *F* = 16.212, *p* = 0.0001). Habitat appeared to be the most important factor, explaining 31.71% of variation in JSD^1/2^ (*adonis*, *F* = 18.117, *p* = 0.0001). In fact, habitat explained more than twice the variance explained by host species factors (11.95%, *adonis*, *F* = 5.295, *p* = 0.0004). However, the PERMANOVA results could be affected by non-homogeneous dispersion of the data. Indeed, the *A. jamaicensis* samples from the Granby Zoo appeared to be less dispersed in JSD^1/2^ (Fig. 5A), and we found different levels of dispersion by habitat (*betadisper*, *F* = 33.4298, *p* = 0.0001) and by habitat and host species combined (*betadisper*, *F* = 7.0628, *p* = 0.0023), but not for host species alone (betadisper, *F* = 3.5063, *p* = 0.0703). Nevertheless, the ordination clearly supports a clustering pattern that confirms the importance of habitat and host species factors combined.

Repeating the same analysis on weighted UniFrac distances yielded similar PERMANOVA results (habitat and host species together: 45.82%, *adonis*, *F* = 16.066, *p* = 0.0001; habitat 32.9%, *adonis*, *F* = 19.1220, *p* = 0.0001; host species: 7.2%, *adonis*, *F* = 3.0131, *p* = 0.0340). However, the UniFrac distances did not vary significantly in dispersion by habitat (*betadisper*, *F* = 1.6594, *p* = 0.2077) or habitat and host species combined (*betadisper*, *F* = 0.2244, *p* = 0.7989). Different species were differently dispersed (*betadisper*, *F* = 8.0426, *p* = 0.0081). Results based on Weighted UniFrac confirm that habitat and host species are acting together to shape the skin microbiome of two species of neotropical bats living in distinct habitats. Habitat appears to be the major driver of microbiome community structure, with a more subtle but significant role of host species.

## Discussion

The skin microbiome is a first line of defense against pathogens (Grice & Segre, 2011). However, the skin microbiome is still poorly investigated in many animals even though it could play a role in disease outcomes. Investigating the fundamental sources of variation in the skin microbiome is therefore a critical step toward understanding its role in health and diseases, and in hope of eventually deploying microbiome-based therapies against wildlife pathogens. This study explored the relative influence of endogenous and exogenous factors (i.e., host species and habitat) in shaping the skin microbiome of two species of frugivorous captive bats. However, the skin microbiome influence is expected to be different in wild populations with respect to bats living in captivity (Becker et al., 2014; Loudon et al., 2014; Cheng et al., 2015). Indeed, captive bats are restricted to a limited area, whereas wild individuals are usually exposed to significant environmental variation when foraging and roosting in nature. The environmental stability of the caves in which captive bats are living could in turn explain the relative importance of the habitat on the skin microbiome with respect to host species in our results.

The species of bats under study were found to have skin-associated microbial communities similar to those characterized in other mammalian orders such as carnivores (dog), marsupials (Tasmanian devil), and primates (human) (Hoffmann et al., 2014; Cheng et al., 2015). Whereas the most abundant bacterial phyla (i.e., Actinobacteria, Proteobacteria, Firmicutes, Cyanobacteria, Bacteroidetes and Fusobacteria) were represented in all of the mammals investigated, relative abundances sometimes differed between species. Namely, Actinobacteria is the most abundant phylum in bat and human profiles alike, Grice & Segre (2011) and Oh et al. (2012), but only the third most abundant in dogs and Tasmanian devils (Hoffmann et al., 2014; Cheng et al., 2015). Such differences could be explained by host species, sampling site on the skin, or habitat variation. Yet, captive neotropical bats were also found to harbor a higher proportion of Cyanobacteria on their skin, a phylum already identified in the gut microbiome of bats (Phillips et al., 2012), particularly in wild populations of *C. perspicillata* (Carrillo-Araujo et al., 2015). The presence of this taxon is suggested to be attributable to the cave habitat, because this type of moist environment is known to be suitable for the establishment of Cyanobacteria (e.g., at the cave entrance where light is available) (Albertano, 2012).

We found a predominant influence of habitat, with a minor but significant role of host species in shaping the microbiome. Specifically, the two cohabitating species, *A. jamaicensis* and *C. perspicillata*, appeared to share more similar skin microbiomes, in terms of composition and diversity, than member of same species *A. jamaicensis* from different habitats. In such gregarious animals (Porter, 1978; Williams, 1986; Ortega & Arita, 1999), individuals inhabiting a single cave are prone to contact with one another, such that microbial transfer is facilitated in captivity, both directly and indirectly. In addition, sharing the same habitat with identical environmental conditions logically incur similar constraints on the skin microbiome of otherwise different host species. Contrary to previous studies on amphibians, which showed significant differences among species cohabiting in the same habitat (McKenzie et al., 2012; Kueneman et al., 2014; Walke et al., 2014), habitat appears to be the main factor acting on the skin microbiome in bats, at least in captivity. Considering that host species and other endogenic factors act in combination with the habitat to drive the skin microbiome structure, we suggest that bat populations could differ in disease susceptibility depending on their immediate environment, as well as the species involved.

Our definition of environmental factors (habitats) is based on a comparison of two different zoos, which could differ in other confounding factors. Some of these confounding factors can be excluded. For example, diet was the same in the two zoos, and the bat colonies were established at the same time (both in 1992). Therefore, diet and time in captivity can be safely excluded as possible confounders of environment. Other factors did differ between zoos. For example, temperature was 26 °C all year long at the Granby Zoo and ranged from 22–26 in the Biodôme. Therefore, we include temperature as part of what we consider to be “environmental effects.” The sex ratio also differed between zoos, with one zoo consisting entirely of males and the other primarily female. However, based on beta diversity analyses, we found no apparent differences in community composition between sexes. Nevertheless, we cannot completely exclude environmental effects being confounded with sex. Sex is known to influence the skin microbiome in humans (Fierer et al., 2008; Ying et al., 2015), and it has been hypothesized that differences in hormone production and metabolism may affect the skin microbiome composition (e.g., pH and sebum production) (Giacomoni, Mammone & Teri, 2009). However, such differences are not always detectable, especially for body sites that do not exhibit sexual differences (e.g., dry sites vs. moist sites) (Oh et al., 2012). In our study, bats were swabbed on the back and the wings—sites which are unlikely to experience sexual dimorphism. Therefore, differences between zoos likely represent environmental factors (e.g., differences in temperature, humidity and environmental bacteria), although we cannot completely exclude a confounding influence of sex.

The predominant effect of the environment (habitat) in shaping the skin microbiome of bats provides both risks and benefits to the host in the face of pathogens. On the negative side, because the bat skin microbiome varies more according to habitat, it might be highly susceptible to invasion by pathogens such as the *Pd* fungus. On the positive side, the skin microbiome could be manipulated with probiotics. This observation suggests that previously considered probiotic (anti-fungal) bacteria such as *Pseudomonas* (Hoyt et al., 2015) and *Rhodococcus rhodochrous* strain DAP96253 (Cornelison et al., 2014) could be introduced directly into bat habitats to ease their implantation in skin communities. Probiotics could therefore represent a promising management tool against pathogens like * Pd*. Of course, such management tools would have to be validated in the relevant host species and environments.

Our investigation of the skin microbiome of two neotropical species of bats living in controlled habitats revealed the combined influence of endogenous and exogenous factors. These results show that the captive bat skin microbiome is shaped both by habitat and host species. Going forward, it will be important to extend our results to additional bat species living in captivity and to wild populations of bats. In bats and other mammals, the skin microbiome has the potential to become an important tool for population health, conservation and management.

##  Supplemental Information

10.7717/peerj.2430/supp-1 Figure S1 Non-metric multidimensional scaling of beta diversity of bat skin microbiome samples and negative controls (A) Non-metric multidimentional scaling of JSD^1/2^ of bat skin microbiome composition. Each point represents a single microbiome sample. 2D stress = 0.09. (B) Non-metric multidimentional scaling of weighted UniFrac distances among bat skin microbiomes. 2D stress = 0.07.

10.7717/peerj.2430/supp-2 Table S1 OTU table presenting the abundance of different bacterial taxa in each sample table, along with the Greengenes ID list associated with the taxonomy

10.7717/peerj.2430/supp-3 Table S2 Complete list of taxa and relative abundance at the phylum, class, order, family and genus level for each group of bats

## Additional Information and Declarations

### Data Availability

The following information was supplied regarding data availability: Figshare Sequencing data DOI: 10.6084/m9.figshare.3428159; Figshare Metadata DOI: 10.6084/m9.figshare.3206668.

## References

[ref-1] Albertano P, Whitton BA (2012). Cyanobacterial biofilms in monuments and caves. Ecology of cyanobacteria II.

[ref-2] Anderson MJ (2001). A new method for non-parametric multivariate analysis of variance. Austral Ecology.

[ref-3] Anderson MJ (2006). Distance-based tests for homogeneity of multivariate dispersions. Biometrics.

[ref-4] Anderson MJ, Walsh DCI (2013). PERMANOVA, ANOSIM, and the Mantel test in the face of heterogeneous dispersions: what null hypothesis are you testing?. Ecological Monographs.

[ref-5] Arita HT, Vargas JA (1995). Natural history, interspecific association, and incidence of the cave bats of Yucatan, Mexico. The Southwestern Naturalist.

[ref-6] Barker GM (2002). Phylogenetic diversity: a quantitative framework for measurement of priority and achievement in biodiversity conservation. Biological Journal of the Linnean Society.

[ref-7] Becker MH, Richards-Zawacki CL, Gratwicke B, Belden LK (2014). The effect of captivity on the cutaneous bacterial community of the critically endangered Panamanian golden frog (*Atelopus zeteki*). Biological Conservation.

[ref-8] Belden LK, Harris RN (2007). Infectious diseases in wildlife: the community ecology context. Frontiers in Ecology and the Environment.

[ref-9] Brucker RM, Harris RN, Schwantes CR, Gallaher TN, Flaherty DC, Lam BA, Minbiole KPC (2008). Amphibian chemical defense: antifungal metabolites of the microsymbiont *Janthinobacterium lividum* on the salamander *Plethodon cinereus*. Journal of Chemical Ecology.

[ref-10] Caporaso JG, Kuczynski J, Stombaugh J, Bittinger K, Bushman FD, Costello EK, Fierer N, González Peña A, Goodrich JK, Gordon JI, Huttley GA, Kelley ST, Knights D, Koenig JE, Ley RE, Lozupone CA, Mcdonald D, Muegge BD, Pirrung M, Reeder J, Sevinsky JR, Turnbaugh PJ, Walters WA, Widmann J, Yatsunenko T, Zaneveld J, Knight R (2010). QIIME allows analysis of high-throughput community sequencing data. Nature Methods.

[ref-11] Caporaso JG, Lauber CL, Walters WA, Berg-Lyons D, Lozupone CA, Turnbaugh PJ, Fierer N, Knight R (2011). Global patterns of 16S rRNA diversity at a depth of millions of sequences per sample. Proceedings of the National Academy of Sciences of the United States of America.

[ref-12] Carrillo-Araujo M, Taş N, Alcántara-Hernández RJ, Gaona O, Schondube JE, Medellín RA, Jansson JK, Falcón LI (2015). Phyllostomid bat microbiome composition is associated to host phylogeny and feeding strategies. Frontiers in Microbiology.

[ref-13] Cheng Y, Fox S, Pemberton D, Hogg C, Papenfuss AT, Belov K (2015). The Tasmanian devil microbiome—implications for conservation and management. Microbiome.

[ref-14] Cloutier D, Thomas DW (1992). Carollia perspicillata. Mammalian Species.

[ref-15] Cornelison CT, Keel MK, Gabriel KT, Barlament CK, Tucker TA, Pierce GE, Crow SA (2014). A preliminary report on the contact-independent antagonism of *Pseudogymnoascus destructans* by *Rhodococcus rhodochrous* strain DAP96253. BMC Microbiology.

[ref-16] DeSantis TZ, Hugenholtz P, Larsen N, Rojas M, Brodie EL, Keller K, Huber T, Dalevi D, Hu P, Andersen GL (2006). Greengenes, a chimera-checked 16S rRNA gene database and workbench compatible with ARB. Applied and Environmental Microbiology.

[ref-17] Edgar RC (2010). Search and clustering orders of magnitude faster than BLAST. Bioinformatics.

[ref-18] Fierer N, Hamady M, Lauber CL, Knight R (2008). The influence of sex, handedness, and washing on the diversity of hand surface bacteria. Proceedings of the National Academy of Sciences of the United States of America.

[ref-19] Fuglede B, Topsøe F (2004). Jensen–Shannon divergence and Hilbert space embedding.

[ref-20] Gargas A, Trest MT, Christensen M, Volk TJ, Blehert DS (2009). *Geomyces destructans* sp. nov. associated with bat white-nose syndrome. Mycotaxon.

[ref-21] Giacomoni P, Mammone T, Teri M (2009). Gender-linked differences in human skin. Journal of Dermatological Science.

[ref-22] Grice EA, Segre JA (2011). The skin microbiome. Nature Reviews Microbiology.

[ref-23] Haegeman B, Hamelin J, Moriarty J, Neal P, Dushoff J, Weitz JS (2013). Robust estimation of microbial diversity in theory and in practice. The ISME Journal.

[ref-24] Hill MO (1973). Diversity and evenness: a unifying notation and its consequences. Ecology.

[ref-25] Hoffmann AR, Patterson AP, Diesel A, Lawhon SD, Ly HJ, Stephenson CE, Mansell J, Steiner JM, Dowd SE, Olivry T, Suchodolski JS (2014). The skin microbiome in healthy and allergic dogs. PLoS ONE.

[ref-26] Holm S (1979). A simple sequentially rejective multiple test procedure. Scandinavian Journal of Statistics.

[ref-27] Hoyt JR, Cheng TL, Langwig KE, Hee MM, Frick WF, Kilpatrick AM (2015). Bacteria isolated from bats inhibit the growth of *Pseudogymnoascus destructans*, the causative agent of white-nose syndrome. PLoS ONE.

[ref-28] Kruskal JB (1964). Multidimensional scaling by optimizing goodness of fit to a nonmetric hypothesis. Psychometrika.

[ref-29] Kruskal WH, Wallis WA (1952). Use of ranks in one-criterion variance analysis. Journal of the American Statistical Association.

[ref-30] Kueneman JG, Parfrey LW, Woodhams DC, Archer HM, Knight R, McKenzie VJ (2014). The amphibian skin-associated microbiome across species, space and life history stages. Molecular Ecology.

[ref-31] Kunz TH, Fenton MB (2003). Bat ecology.

[ref-32] Lorch JM, Meteyer CU, Behr MJ, Boyles JG, Cryan PM, Hicks AC, Ballmann AE, Coleman J, Redell DN, Reeder DM, Blehert DS (2011). Experimental infection of bats with *Geomyces destructans* causes white-nose syndrome. Nature.

[ref-33] Loudon AH, Woodhams DC, Parfrey LW, Archer H, Knight R, McKenzie V, Harris RN (2014). Microbial community dynamics and effect of environmental microbial reservoirs on red-backed salamanders (*Plethodon cinereus*). The ISME Journal.

[ref-34] Lozupone CA, Hamady M, Kelley ST, Knight R (2007). Quantitative and qualitative *β* diversity measures lead to different insights into factors that structure microbial communities. Applied and Environmental Microbiology.

[ref-35] Lozupone CA, Knight R (2005). UniFrac: a new phylogenetic method for comparing microbial communities. Applied and Environmental Microbiology.

[ref-36] Lozupone CA, Lladser ME, Knights D, Stombaugh J, Knight R (2011). UniFrac: an effective distance metric for microbial community comparison. The ISME Journal.

[ref-37] McCoy CO, Matsen IV FA (2013). Abundance-weighted phylogenetic diversity measures distinguish microbial community states and are robust to sampling depth. PeerJ.

[ref-38] McKenzie VJ, Bowers RM, Fierer N, Knight R, Lauber CL (2012). Co-habiting amphibian species harbor unique skin bacterial communities in wild populations. The ISME Journal.

[ref-39] McMurdie PJ, Holmes S (2013). Phyloseq: an R package for reproducible interactive analysis and graphics of microbiome census data. PLoS ONE.

[ref-40] McMurdie PJ, Holmes S (2014). Waste not, want not: why rarefying microbiome data is inadmissible. PLoS Computational Biology.

[ref-41] Minnis AM, Lindner DL (2013). .Phylogenetic evaluation of *Geomyces* and allies reveals no close relatives of *Pseudogymnoascus destructans*, comb. nov., in bat hibernacula of eastern North America. Fungal Biology.

[ref-42] Moeller AH, Peeters M, Ndjango J-B, Li Y, Hahn BH, Ochman H (2013). Sympatric chimpanzees and gorillas harbor convergent gut microbial communities. Genome Research.

[ref-43] Morrison DW (1979). Apparent male defense of tree hollows in the fruit bat, *Artibeus jamaicensis*. Journal of Mammalogy.

[ref-44] Ochman H, Worobey M, Kuo C-H, Ndjango J-BN, Peeters M, Hahn BH, Hugenholtz P (2010). Evolutionary relationships of wild hominids recapitulated by gut microbial communities. PLoS Biology.

[ref-45] Oh J, Conlan S, Polley EC, Segre JA, Kong HH (2012). Shifts in human skin and nares microbiota of healthy children and adults. Genome Medicine.

[ref-46] Oksanen J, Blanchet G, Kindt R, Legendre P, Minchin PR, O’Hara RB, Simpson GL, Solymos P, Stevens M, Wagner H (2015). http://CRAN.R-project.org/package=vegan.

[ref-47] Ortega J, Arita HT (1999). Structure and social dynamics of harem groups in *Artibeus jamaicensis* (Chiroptera: Phyllostomidae). Journal of Mammalogy.

[ref-48] Ortega J, Castro-Arellano I (2001). Artibeus jamaicensis. Mammalian Species.

[ref-49] Phillips CD, Phelan G, Dowd SE, McDonough MM, Ferguson AW, Hanson JD, Siles L, Ordóñez-Garza N, San Francisco M, Baker RJ (2012). Microbiome analysis among bats describes influences of host phylogeny, life history, physiology and geography. Molecular Ecology.

[ref-50] Porter FL (1978). Roosting patterns and social behavior in captive *Carollia perspicillata*. Journal of Mammalogy.

[ref-51] Preheim SP, Perrotta AR, Friedman J, Smilie C, Brito I, Smith MB, Alm EJ, Edward FD (2013a). Computational methods for high-throughput comparative analyses of natural microbial communities. Methods in enzymology.

[ref-52] Preheim SP, Perrotta AR, Martin-Platero AM, Gupta A, Alm EJ (2013b). Distribution-based clustering: using ecology to refine the operational taxonomic unit. Applied and Environmental Microbiology.

[ref-53] Price MN, Dehal PS, Arkin AP (2010). FastTree 2: approximately maximum-likelihood trees for large alignments. PLoS ONE.

[ref-54] R Developement Core Team (2015).

[ref-55] Roeselers G, Mittge EK, Stephens WZ, Parichy DM, Cavanaugh CM, Guillemin K, Rawls JF (2011). Evidence for a core gut microbiota in the zebrafish. The ISME Journal.

[ref-56] Romano-Bertrand S, Licznar-Fajardo P, Parer S, Jumas-Bilak E (2015). Impact de l’environnement sur les microbiotes: focus sur l’hospitalisation et les microbiotes cutanés et chirurgicaux. Revue Francophone des Laboratoires.

[ref-57] Roth RR, James WD (1988). Microbial ecology of the skin. Annual Review of Microbiology.

[ref-58] Segata N, Izard J, Waldron L, Gevers D, Miropolsky L, Garrett WS, Huttenhower C (2011). Metagenomic biomarker discovery and explanation. Genome Biology.

[ref-59] Song SJ, Lauber C, Costello EK, Lozupone CA, Humphrey G, Berg-Lyons D, Caporaso JG, Knights D, Clemente JC, Nakielny S, Gordon JI, Fierer N, Knight R (2013). Cohabiting family members share microbiota with one another and with their dogs. eLife.

[ref-60] Turner GG, Reeder DM, Coleman JTH (2011). A five-year assessment of mortality and geographic spread of white-nose syndrome in North American bats, with a look at the future: update of white-nose syndrome in bats. Bat Research News.

[ref-61] Tzeng T-D, Pao Y-Y, Chen P-C, Weng FC-H, Jean WD, Wang D (2015). Effects of host phylogeny and habitats on gut microbiomes of oriental river prawn (*Macrobrachium nipponense*). PLoS ONE.

[ref-62] US Fish & Wildlife Service (2012). North American bat death toll exceeds 5.5 million from white-nose syndrome. http://www.batcon.org/pdfs/USFWS_WNS_Mortality_2012_NR_FINAL.pdf.

[ref-63] Vellend M, Cornwell WK, Magnuson-Ford K, Mooers AØ, Magurran AE (2011). Measuring phylogenetic biodiversity. Biological diversity: frontiers in measurement and assessment.

[ref-64] Voigt CC, Kingston T (2016). Bats in the anthropocene: conservation of bats in a changing world.

[ref-65] Walke JB, Becker MH, Loftus SC, House LL, Cormier G, Jensen RV, Belden LK (2014). Amphibian skin may select for rare environmental microbes. The ISME Journal.

[ref-66] Wilcoxon F (1945). Individual comparisons by ranking methods. Biometrics Bulletin.

[ref-67] Williams CF (1986). Social organization of the bat, *Carollia perspicillata* (Chiroptera: Phyllostomidae). Ethology.

[ref-68] Ying S, Zeng DN, Chi L, Tan Y, Galzote C, Cardona C, Lax S, Gilbert J, Quan ZX (2015). The influence of age and gender on skin-associated microbial communities in urban and rural human populations. PLoS ONE.

