# Supplementary figures and images for: Environment and host species shape the skin microbiome of captive neotropical bats
Source: PeerJ. 2016 Sep 20;4:e2430. doi: 10.7717/peerj.2430 (PMC5036103; doi:10.7717/peerj.2430)

**A**

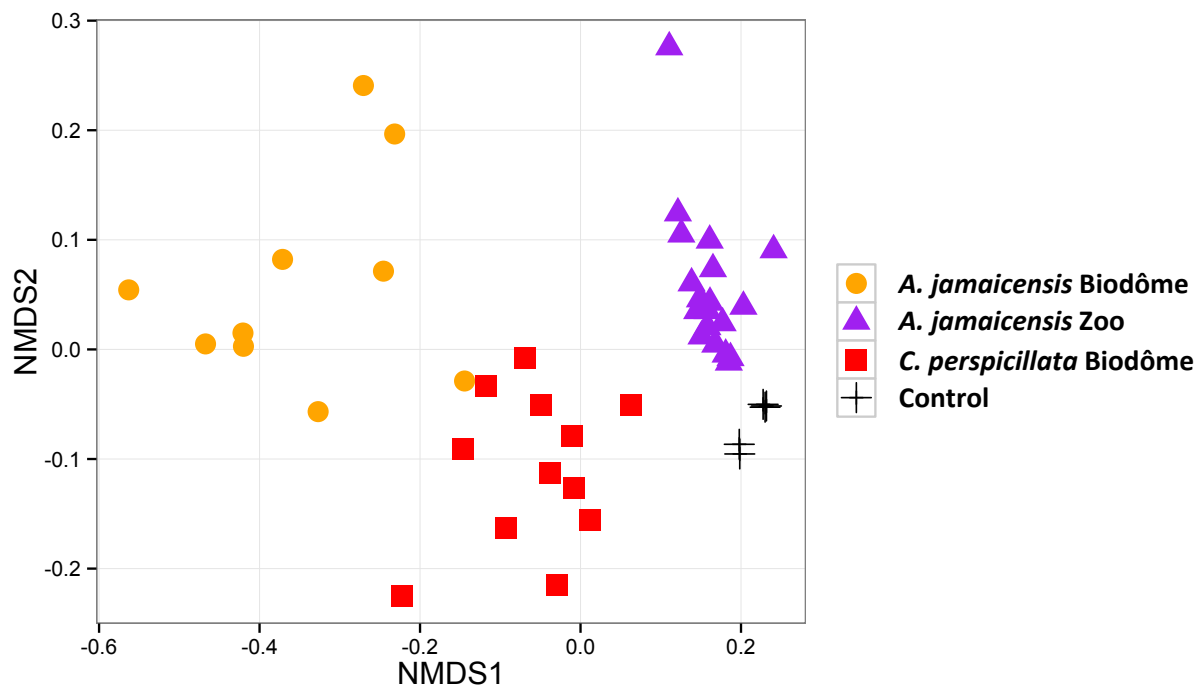

**B**

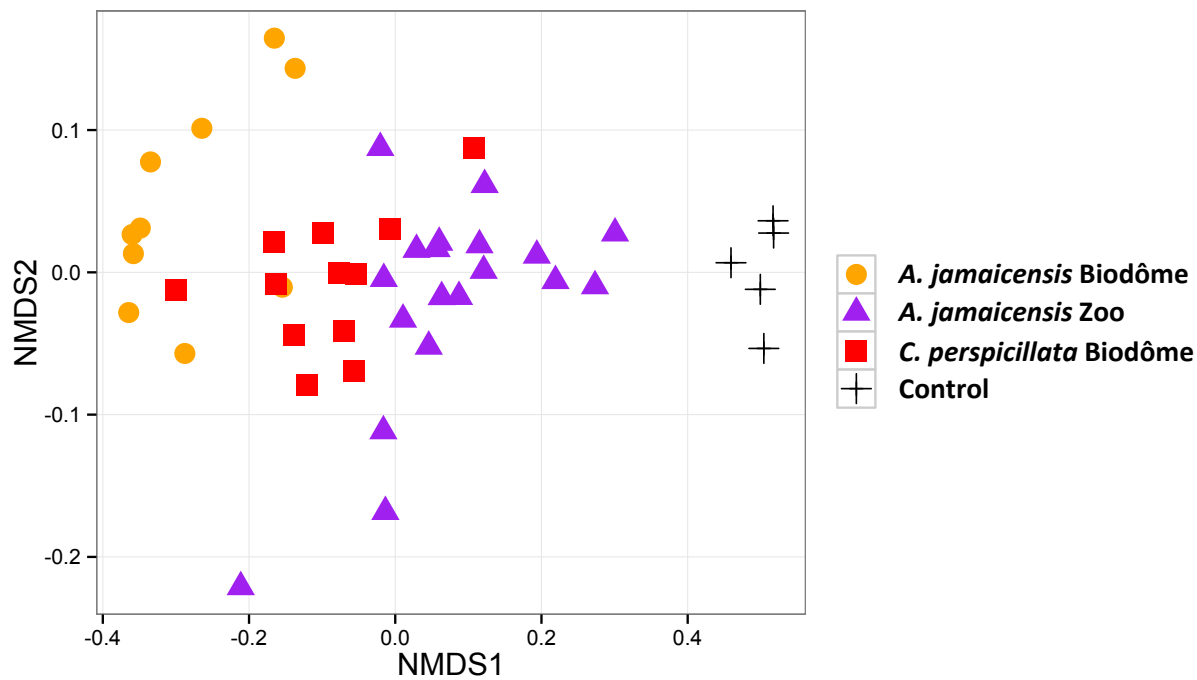

Supplement: Figure S1 — (A) Non-metric multidimentional scaling of JSD1/2 of bat skin microbiome composition. Each point represents a single microbiome sample. 2D stress = 0.09. (B) Non-metric multidimentional scaling of weighted UniFrac distances among bat skin microbiomes. 2D stress = 0.07. [file peerj-04-2430-s001.pdf]
